# Conservative Management of a Retained Appendicolith via Percutaneous Drainage Following Laparoscopic Appendectomy: A Case Report

**DOI:** 10.7759/cureus.111977

**Published:** 2026-07-03

**Authors:** Kodai Takemori, Tomoya Hagiwara, Masachika Kitajima, Keiji Inoue

**Affiliations:** 1 Department of Surgery, Saga National Hospital, Saga City, JPN; 2 Department of Surgery, Nagasaki Goto Chuoh Hospital, Goto City, JPN

**Keywords:** intra-abdominal abscess, laparoscopic appendectomy, percutaneous drainage, retained appendicolith, spontaneous disappearance

## Abstract

Retained appendicolith is an uncommon but recognized complication following laparoscopic appendectomy that can result in the formation of an intra-abdominal abscess. Although surgical retrieval of the stone is generally recommended to prevent recurrence, the optimal management strategy remains controversial. We report a case of a 16-year-old male patient who underwent an urgent laparoscopic appendectomy for acute complicated appendicitis. Intraoperative injury to the appendix resulted in the spillage of the appendicoliths into the peritoneal cavity. The postoperative course was initially uneventful. On postoperative day 3, the patient was asymptomatic with a normalized white blood cell count; consequently, antibiotics were discontinued, and the intra-abdominal drain was removed following confirmed normal output. The patient was discharged on postoperative day 6. Two days after discharge, the patient presented to the outpatient clinic with abdominal pain and purulent discharge from the drain site, leading to readmission. A CT scan revealed an intra-abdominal abscess caused by a retained appendicolith. CT-guided percutaneous drainage was performed, which led to the immediate resolution of abdominal pain within 24 hours. The abscess cavity was irrigated daily with normal saline, and the drainage fluid remained mostly clear from the first morning after insertion. Four days after the drainage tube placement, retrieval of the retained appendicolith was attempted using an 11-Fr angiographic sheath inserted through the existing drainage tract under fluoroscopic guidance. The stone (approximately 3 mm in diameter) could not be removed despite repeated irrigation. Given the small size of the appendicolith and adequate infection control achieved with drainage and irrigation, conservative management was chosen. The potential risks were thoroughly explained to the patient and his caregivers, who confirmed their understanding. A follow-up CT scan at six months after surgery revealed no intra-abdominal abscess, and the appendicolith had disappeared. Although the removal of retained appendicoliths is generally advocated, careful observation after sufficient drainage may be a reasonable and safe option for certain patients.

## Introduction

Appendicoliths are commonly detected in patients with acute appendicitis and have been reported to increase the risk of progression to complicated appendicitis [1,2]. During urgent laparoscopic appendectomy, appendicoliths may inadvertently spill into the peritoneal cavity, resulting in intra-abdominal abscess formation. The management of intra-abdominal abscesses caused by retained appendicoliths remains controversial and clinically challenging [3,4]. We report a case of a young male patient who developed an intra-abdominal abscess secondary to retained appendicoliths following urgent laparoscopic appendectomy. The abscess was managed conservatively, resulting in a favorable clinical outcome.

## Case presentation

A 16-year-old male patient with no significant medical history presented with abdominal pain and fever. He was initially diagnosed with a urinary tract infection at a local clinic and treated with oral antibiotics; however, his symptoms persisted. Four days after symptom onset, he was referred to our hospital. Physical examination revealed right lower quadrant tenderness without signs of peritoneal irritation. Enhanced computed tomography (CT) revealed a swollen appendix with several high-density areas consistent with appendicoliths. The appendiceal wall was partially discontinuous, suggesting leakage of intraluminal contents (Figure 1). Based on these findings, the patient was diagnosed with perforated appendicitis and underwent an urgent laparoscopic appendectomy.

**Figure 1:**
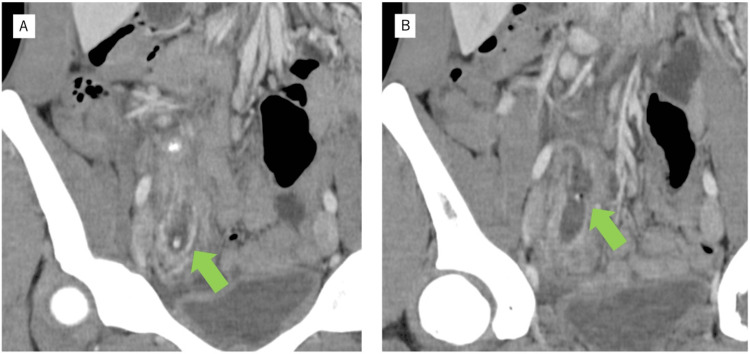
Computed tomography (CT) findings A, B: contrast-enhanced CT showing an enlarged and perforated appendix containing multiple appendicoliths (arrow).

Intraoperatively, the appendix was found to be severely inflamed, pre-gangrenous, and enveloped by the omentum, forming a small surrounding abscess. Although no obvious pre-existing perforation site was identified, the appendix was accidentally perforated during intraoperative manipulation, resulting in the spillage of appendicoliths into the peritoneal cavity. The spilled appendicoliths were retrieved as thoroughly as possible. The appendix was resected after double ligation of the base and retrieved using a specimen bag, and a 15-Fr multi-channel drain was placed in the rectovesical pouch. Ascites culture collected intraoperatively yielded *Citrobacter freundii* (1+), which was highly susceptible to ampicillin/sulbactam.

The postoperative course was initially uneventful. On postoperative day 3, the patient was asymptomatic with a normalized white blood cell count; consequently, antibiotics (ampicillin/sulbactam) were discontinued, and the intra-abdominal drain was removed following confirmed normal output. No oral antibiotics were used after stopping the intravenous antibiotics. The patient was discharged on postoperative day 6.

Two days after discharge, the patient presented to the outpatient clinic with abdominal pain and purulent discharge from the drain site, leading to readmission. A CT scan revealed a localized intra-abdominal abscess measuring 25 mm in maximum diameter, located contiguous with the previous drain site. Notably, a high-density structure consistent with the spilled appendicolith was confirmed to be contained directly within the abscess cavity (Figure 2). Intravenous antibiotics (ampicillin/sulbactam) were restarted, and CT-guided percutaneous drainage was performed through the previous drain site on the day of readmission, which led to the immediate resolution of abdominal pain within 24 hours. The abscess cavity was irrigated daily with 10-20 ml of normal saline, and the drainage fluid remained mostly clear from the first morning after insertion. The pus culture collected from the abscess subsequently yielded *Streptococcus anginosus* (3+)and *Fusobacterium necrophorum* (3+), both of which confirmed high susceptibility to ampicillin/sulbactam.

**Figure 2:**
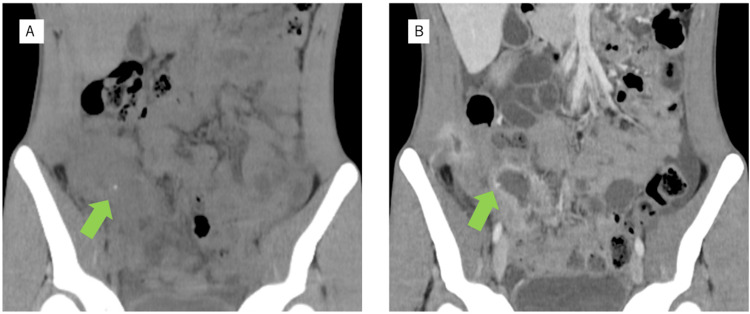
Imaging findings A: non-contrast computed tomography (CT) demonstrating a high-density lesion, approximately 3 mm in diameter, suggestive of a retained appendicolith (arrow); B: contrast-enhanced CT revealing a retained appendicolith within an encapsulated abscess cavity, measuring 25 mm in maximum diameter (arrow).

Four days after percutaneous drainage, following consultation with an experienced interventional radiologist, we attempted to retrieve the stone using an 11-Fr angiographic sheath inserted through the existing drainage tract under fluoroscopic guidance (Figure 3). Although the appendicolith was small (approximately 3 mm in diameter), it remained stationary despite repeated saline irrigation, and retrieval was unsuccessful. After thorough informed consent was obtained from the patient and his guardian, conservative management was selected through shared decision-making.

**Figure 3:**
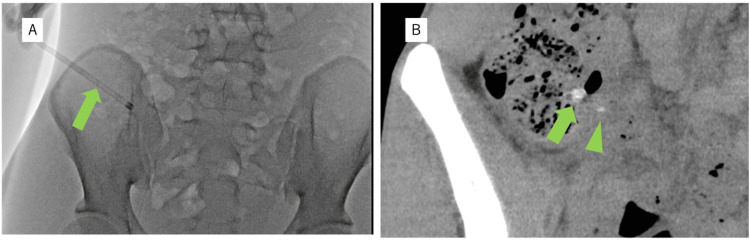
Imaging findings A: an 11-Fr angiographic sheath was inserted through the drainage tract, and repeated irrigation was performed (arrow); B: computed tomography after the removal attempt confirmed persistence of the retained appendicolith, measuring approximately 3 mm in diameter (arrowhead), the arrow indicates the inserted sheath.

The patient remained asymptomatic without recurrence. A follow-up CT performed six months after the surgery demonstrated no intra-abdominal abscess and disappearance of the appendicolith.

## Discussion

In the present case, urgent laparoscopic appendectomy was selected as the initial treatment. The patient subsequently developed a postoperative intra-abdominal abscess associated with a retained appendicolith. Although this complication might have been avoided if nonoperative management (NOM) had been successful, treatment failure may worsen the inflammation, potentially necessitating extended resection or conversion to open surgery. Furthermore, the presence of an appendicolith is a well-recognized risk factor for failure of NOM and recurrence [5-7]. Additionally, our institution is located on a remote island, where immediate surgical intervention may not always be readily available in the event of sudden clinical deterioration. Therefore, urgent laparoscopic appendectomy was considered an appropriate initial treatment in the present case.

Retained appendicoliths are recognized but relatively rare complications of laparoscopic appendectomy, particularly in complicated appendicitis [3,4]. Appendicoliths may serve as a persistent nidus of infection, leading to chronic infection and delayed recurrent intra-abdominal abscesses [3,8,9]. Although surgical retrieval has traditionally been recommended to prevent recurrence, minimally invasive percutaneous extraction techniques have also been reported [4,10]. In the present case, we attempted minimally invasive retrieval using an 11-Fr angiographic sheath. Despite repeated irrigation, the appendicolith remained stationary and could not be removed. This unsuccessful retrieval may have been attributable to the timing of the procedure. Four days after percutaneous drainage, the appendicolith had presumably become firmly embedded within inflammatory granulation tissue. In retrospect, earlier intervention immediately after the initial drainage might have been more effective.

Despite unsuccessful retrieval of the appendicolith, conservative treatment rendered a successful outcome for the patient without surgical intervention. Several factors may explain the absence of a recurrence. First, although definitive size thresholds for conservative management are not established, Singh et al. reported that four out of six patients with retained appendicoliths measuring 4-10 mm were successfully cured by drainage alone [4]. In our case, the stone was exceptionally small (~3 mm), falling below this reported range and thus limiting its potential to serve as a persistent site of infection. Second, intensive local infection control was achieved through daily irrigation and copious saline lavage during the attempted retrieval. This likely reduced the bacterial burden and inflammatory debris.

The management strategies for retained appendicoliths remain controversial. Some studies suggest that percutaneous drainage alone remains insufficient and that surgical removal is mandatory due to a high long-term failure rate of conservative treatment [8,9,11]. Conversely, other reports have demonstrated successful outcomes with conservative management, including spontaneous disappearance of the appendicolith as in this case [4,12,13]. Given these conflicting data, a definitive long-term prognosis remains unpredictable. Therefore, while conservative treatment may avoid immediate surgical reintervention, it carries potential long-term risks such as chronic infection, late recurrence, and delayed complications. This strategy should not be universally generalized but may be considered a viable, limited option for carefully selected patients who thoroughly understand and accept these unestablished risks, provided that strict, long-term clinical follow-up is guaranteed. Lastly, as this is a single case report, it has limited generalizability and cannot establish a standard of care or prove a definitive causal relationship.

## Conclusions

We report a case of an intra-abdominal abscess caused by retained appendicoliths following emergency surgery for acute complicated appendicitis. Although removal of retained appendicoliths is generally recommended because of the risk of recurrence, careful observation may be a reasonable and beneficial management option in selected cases to avoid repeated surgeries.
